# Resolving latent safety threats identified through in situ simulation: a multicentre mixed-methods study

**DOI:** 10.1186/s41077-025-00401-y

**Published:** 2025-12-08

**Authors:** Jennifer Weller, Kate Fahey-Williams, Kaylene Henderson, Jane Torrie, James Hamill, James Moore, Carlos Campos, Andrew MacCormick

**Affiliations:** 1 Centre for Medical and Health Sciences Education, School of Medicine, University of Auckland, Auckland, New Zealand; 2 Department of Anaesthesia and Perioperative Medicine, Auckland City Hospital, Auckland, New Zealand; 3 Department of Anesthesiology, School of Medicine, University of Auckland, Auckland, New Zealand; 4 Department of Surgery, Starship Hospital, Auckland, New Zealand; 5 Department of Surgery, School of Medicine, University of Auckland, Auckland, New Zealand; 6 Intensive Care Unit, Wellington Hospital, Wellington, New Zealand; 7 Medical Program Directorate, School of Medicine, University of Auckland, Auckland, New Zealand; 8 Department of Surgery, Middlemore Hospital, Auckland, New Zealand

**Keywords:** In situ simulation, Latent safety threats, Patient safety, Quality improvement, Healthcare systems, Clinical governance

## Abstract

**Background:**

In situ simulation can identify latent safety threats in healthcare, yet there has been limited focus on how these threats are subsequently addressed. Adopting a systematic approach to identifying, reporting, and resolving threats found during in situ simulations could enhance clinical safety and system resilience. This study investigated the resolution of safety threats detected through in situ simulation courses in Aotearoa New Zealand hospitals, aiming to quantify resolution rates and examine factors influencing successful resolution.

**Methods:**

This multicentre study used an exploratory sequential mixed-methods design. We collected data on latent safety threats identified after in situ simulations using a structured reporting tool and assessed their resolution three months post-course. Associations between resolution and threat classification, risk assessment score, course type, and hospital size were analysed. Qualitative interviews with hospital simulation convenors explored contextual and experiential factors affecting resolution.

**Results:**

Across 20 courses in 15 hospitals, 278 safety threats were identified at the three-month follow-up, with 28% resolved. Threats involving equipment, environmental layout, and tasks were more often resolved than those related to teamwork or organisational factors. Smaller hospitals showed higher resolution rates; multilevel regression confirmed hospital size and threat classification as significant predictors of resolution. Qualitative thematic analysis of 15 interviews identified five key themes: influence of threat type; motivation to resolve the threat; identifying and communicating the threat; clinician agency within their organisation; and hospital structures and processes to support resolution of identified safety threat. Tangible threats within clinicians’ control were addressed more readily, often through straightforward interventions; conversely, threats requiring cross-departmental collaboration or structural change remained unresolved due to limited authority, time, and institutional support.

**Conclusion:**

While in situ simulation effectively identifies latent safety threats, threat resolution remains limited. Our findings highlight the need to align institutional processes with frontline clinicians’ insights. Effective threat mitigation depends on both threat characteristics and organisational context. To fully realise the opportunity presented by in situ simulation to improve patient safety, healthcare systems must move beyond threat identification to actively support resolution—by empowering clinicians, enabling multidisciplinary collaboration, and embedding clear processes for follow-up and accountability.

**Supplementary Information:**

The online version contains supplementary material available at 10.1186/s41077-025-00401-y.

## Background

Patient care occurs within a complex, dynamic system vulnerable to error, potentially compromising outcomes. Errors can result in adverse events that harm patients and staff or generate system inefficiencies with long-term implications for care delivery. These limitations often stem from identifiable, underlying contributory factors, often referred to as latent safety threats (LSTs).

LSTs may lie dormant within a system over time, only causing adverse consequences for patients or staff when a number of factors combine, which together, overcome inbuilt defences in the system [1]. In situ simulation provides an opportunity to proactively identify these threats by challenging the system, for example, through emergency clinical scenarios, and thus an opportunity to address them before they cause actual harm [2]. While previous studies have demonstrated the effectiveness of in situ simulation in uncovering LSTs [3–6] less attention has been paid to how reported LSTs are acted upon. Some simulation programs have successfully resolved most identified LSTs, whereas others report low resolution rates [7, 8]

A systematic approach to identifying, reporting, and resolving patient safety threats identified during in situ simulations could strengthen clinical safety and system resilience. Gaining insight into the types of threats uncovered, clinicians’ responses, and the factors that influence resolution may promote more effective problem-solving, enable sharing of solutions, and inform structured safety improvement processes.

Building on our earlier work reporting frequencies and classifications of LSTs identified through in situ team training [5], this study aims to quantify threat resolution and explore factors that support or hinder the efforts of local healthcare staff to address them within their clinical environments.

Our research questions were:

1. What proportion of safety threats identified during in situ simulation are resolved at three months, and what factors influence resolution status.
2. How are threats to patient and staff safety identified through in-situ simulation; what are the experiences of clinicians in attempting to resolve these threats; and what factors facilitate or hinder threat resolution.

## Methods

This study received ethical approval from the Auckland Health Research Ethics Committee (Ref AH23016) on 23 July 2021. Written consent was obtained from all participating hospitals and interviewees.

### Context

The study was conducted within NetworkZ, a national multidisciplinary in situ simulation team-training initiative in Aotearoa New Zealand, led and administered by the University of Auckland [9, 10]. Funded by government agencies (the Accident Compensation Corporation, New Zealand District Health Board, and the New Zealand Transport Authority) NetworkZ was established in 2016. To date, approximately 265 courses have engaged over 3,350 clinical staff in half-day simulation-based training.

Each course includes three in situ simulations, structured debriefs, and interactive presentations on teamwork and safety. The teamwork model is based on Salas et al.’s framework, [11] emphasising five key features of effective teams: leadership, adaptability, mutual performance monitoring, back-up behaviour, and team orientation. These are underpinned by: shared mental models, mutual trust and respect, and clear communication. There is a strong emphasis on creating psychological safety for participants and ensuring in situ simulations do not create any hazards for subsequent patients or staff. Consultation with Māori colleagues has embedded cultural safety throughout the course.

Scenarios and resources are tailored to specific clinical areas, with about 30 scenarios available, all based on real cases. Scenarios aim for a high degree of realism, including complete patient notes and laboratory documentation, interactive surgical models, moulage integrated with a Laerdal 3G computerised manikin (Laerdal Medical, Norway), bespoke silicone face masks, and the drugs, disposables, and equipment available locally. Blood products are manufactured in collaboration with the New Zealand Blood Bank and clearly labelled as simulated.

Training occurs in clinical environments such as operating rooms (OR), emergency departments (ED), post-anaesthesia care units (PACU), and radiology. Local convenors—clinicians with educational or managerial roles—lead sessions, document safety threats, and initiate follow-up actions following each course. NetworkZ faculty support local convenors with course delivery and debriefing.

All team members participate in their usual roles, with paramedics, radiographers, blood bank staff, and social workers included as appropriate. An assistant in the room facilitates interaction with simulation equipment. Scenarios unfold as they would in real life—with no attempt to provoke errors or provide misleading information. Before simulations, participants are prompted to consider how the environment affects case management, and these are addressed during debriefs.

### Study design

We employed an explanatory sequential mixed methods design [12], a research design which begins with quantitative data collection and analysis, followed by a qualitative phase to explain and deepen understanding of the initial results. This approach integrates numerical trends with contextual insights for a comprehensive interpretation. Quantitative data were collected from the structured post-course reports and follow-up assessments at three months and underwent statistical analysis. Qualitative data were gathered through semi-structured interviews with local convenors aiming to explain how threats were identified and addressed, and underwent inductive thematic analysis. The two data sets were then merged to provide a comprehensive understanding of safety threat resolution.

### Quantitative study

We analysed safety threats reported by local convenors following NetworkZ courses. The primary outcome was the proportion of threats resolved at three months. We also examined associations between resolution and key variables: threat classification, course location (clinical department), hospital size (by bed number), and risk assessment score [13]. 

### Post-course LST reporting tool

We used the London Protocol and contributing factors framework to categorise the LSTs. The protocol was originally developed to categorise adverse clinical events [14], and we had previous experience with the protocol in an observational study documenting LSTs in in situ training courses [5]. The categories were incorporated into an online-reporting tool with drop down menus for descriptors and subcategories. The tool was piloted in two courses and supported by face-to-face and online training for convenors. It enabled systematic documentation of LSTs and also recorded follow-up actions. (Supplementary material: LST reporting tool Table S1.)

### Sample population

The sample included local convenors from hospitals participating in NetworkZ courses in the OR, ED, or PACU. Due to the exploratory nature of this study, no sample size estimate was undertaken, rather we aimed to include all available post-course report data.

### Data collection

Convenors submitted post-course reports with support from NetworkZ faculty. They were prompted via email at six weeks to submit an interim report and at three months to submit a final report on the resolution status of identified threats. Non-responders received up to three reminders.

### Risk assessment score

Five researchers (JW, JM, JH, JT, KH) independently evaluated each LST by scoring both the severity and the likelihood of recurrence. We used a 2 × 2 matrix, assigning scores from 1 to 5 for both likelihood and severity, following the Healthcare Failure Mode and Effect Analysis (HFMEA) scoring methodology to determine the overall risk level for each threat [13, 15]. Prior to scoring, the group convened to discuss the interpretation of the scoring tool. During the scoring process, the group met on two further occasions to clarify and agree on the scoring approach, address variations in individual scores, and reach consensus on the final ratings.

Scores were grouped into low, moderate, high and extreme risk categories. (Supplementary Table S2). An extreme risk (score of 20–25) would be catastrophic or cause major harm and almost certain or likely to occur, while low risk (score of 1–4) would be associated with minor or minimal harm and would be rare or unlikely to occur. Local clinical staff were not involved in the risk assessment scoring.

### Statistical analysis

Threats were categorised and resolution status was recorded as a binary variable (resolved/not resolved). Using IBM SPSS Statistics (Version 27), we tested the association between our predicted variables (threat classification, course type, risk assessment score, hospital size) and resolution status (binary outcome variable) individually. We used Chi-square test to explore if there was a difference in resolution status within the categorical variables threat classification, course type, and risk assessment score. We use pair-wise comparisons with Bonferroni correction to further investigate any categorical variables which demonstrated a significant difference in resolution status. We used independent-samples t-test to explore the association between hospital size (by number of beds) and resolution status.

To examine the association between the predicted variables and resolution status—while controlling for all relevant factors—we conducted a multilevel logistic regression analysis (a random intercept model was specified, allowing the intercept to vary across courses). We used the conditional pseudo-R² statistic to assess how much variance in the outcome was explained by our model. Because safety threats were grouped within specific courses, the data were not fully independent. To account for this, we first ran an intercept-only model to check for clustering. This model yielded an intraclass correlation coefficient (ICC) of 0.348 and therefore indicated substantial clustering within courses. In other words, safety threats identified within the same course were more similar to each other than to those from other courses. This provided a strong rationale for applying a multilevel regression approach to properly handle the nested structure of the data [16]. 

### Qualitative study

We conducted semi-structured interviews with 15 local convenors between October 2023 and October 2024. Participants were purposively sampled to ensure diversity in geography, hospital size, and professional role. Interviews were conducted by a single researcher (KFW), audio-recorded, professionally transcribed, and analysed using NVivo software (QSR International Pty Ltd. Version 12, 2018). (Supplementary Table S3 - Interview guide).

We reviewed transcripts iteratively and continued recruitment until we reached the point of data saturation, where no new themes or important new ideas were arising, after which we conducted two further interviews.

We followed Braun and Clarke’s thematic analysis approach [17]. Two researchers (JW, KFW) independently reviewed transcripts, developed a coding framework, and refined themes through iterative discussion with the broader research team.

### Data integration

We linked quantitative findings to interview themes, exploring which themes and concepts explained the findings from the quantitative findings on threat resolution and the factors that influenced resolution.

## Results

### Quantitative results

Post-course report data were collected from courses between May 2022 and May 2024, comprising 67 initial post-course reports containing 885 LSTs from 24 hospitals (5 tertiary, 18 regional, 1 rural). We obtained 3-month follow-up data on 278 safety threats from 20 courses (including 13 ED, 4 OR, and 3 PACU courses) at 15 locations (3 tertiary, 11 regional and 1 rural hospitals). (Supplementary Table S4.) At three months, 28% percent of threats were reported as resolved. The threat classification, course type, hospital size and risk assessment score, and the resolution status are shown in Table 1.

**Table 1:** Description of the proportion of LSTs that were resolved versus not resolved by threat classification, course type, risk assessment score, and hospital size

		Total *N* LSTs	*N* LSTs resolved (%)	*N* LSTs not resolved (%)	Statistics	
					χ^2^	*p*
Overall		278	78 (28.1)	200 (71.9)		
Threat Classification	Teamwork	77	9 (11.7)	68 (88.3)		
	Environment	33	14 (42.4)	19 (57.6)		
	Equipment	46	22 (47.8)	24 (52.2)		
	Staff	47	11 (23.4)	36 (76.6)		
	Tasks	51	14 (27.4)	37 (72.6)		
	Organisational	19	6 (31.6)	13 (68.4)		
	Other	5	2 (40.0)	3 (60.0)		
					23.49	< 0.001
Course Type	ED	221	62 (28.1)	159 (71.9)		
	OR	30	6 (20.0)	24 (80.0)		
	PACU	27	10 (37.0)	17 (62.9)		
					2.04	0.360
Risk Assessment Score	Low	0	0 (0.0)	0 (0.0)		
	Moderate	129	33 (25.6)	96 (74.4)		
	High	130	39 (30.0)	91 (70.0)		
	Extreme	19	6 (31.6)	13 (68.4)		
					0.75	0.687
		**Mean no. beds (sd)**	**Mean no. beds resolved (sd)**	**Mean no. beds not resolved (sd)**	**Statistics**	
					***t***	***p***
Hospital size (bed no.)		407.1 (361.3)	289.6 (288.9)	452.8 (376.7)	3.45	< 0.001

*ED* Emergency Department, *OR* Operating Room, *PACU* Post Anaesthesia Care Unit, *N* Number, *no*. number, *sd* standard deviation, *χ2* Chi squared

There was a significant univariate association between resolution status at three months and threat classification (*p* < 0.001). Pairwise comparisons indicated that safety threats classified as teamwork factors were significantly less likely to be resolved compared to safety threats classified as environment (*p* < 0.001) or equipment factors (*p* = 0.017). There were no other significant interactions between threat classification factors. We found no significant relationship between resolution status and either of course type or risk assessment score (Table 1).

We found a difference in hospital size (bed number) for resolved safety threats (*Mean* = 289.63 beds, *SD* = 288.94) versus not resolved LSTs (*Mean* = 452.83 beds, *SD* = 376.71), which reached significance (*Mean difference* = 163.2 beds, 95% CI [70.06, 256.34], *p* < 0.001). This suggests safety threats were more likely to be resolved in smaller hospitals compared to larger hospitals (Table 1).

In summary, in the univariate analysis, threat classification and hospital size appeared as the only significant predictors of safety threat resolution.

### Multilevel regression analysis

We conducted the multilevel regression analysis using the two variables that showed a significant association with resolution status: hospital size and threat classification. Hospital size was treated as a continuous variable (based on the number of beds) at the course level, while threat classification was included as a categorical variable at the threat level. For threat classification, “teamwork factors” was used as the reference as it was the most frequently selected classification.

(Table 2).

**Table 2:** Multilevel logistic regression output for hospital size (bed number), threat classification, risk assessment score, and course type as predictors of LST resolution status

		β (std. error)	95% CI β [lower, upper]	t	*p*	Odds Ratio	95% CI odds ratio [lower, upper]
	Intercept	−1.89 (0.69)	[−3.25, −0.53]	−2.74	0.007	0.15	[0.39, 0.59]
	Hospital size (bed no.)	−0.002 (0.0011)	[−0.004,−1.09E^− 5^]	−1.98	0.049	0.998	[0.996, 1.0]
Threat Classification	Environment	2.12 (0.59)	[0.96, 3.28]	3.61	< 0.001	8.34	[2.62, 26.51]
	Equipment	2.26 (0.57)	[1.14, 3.37]	3.98	< 0.001	9.56	[3.13, 29.19]
	Organisational	1.23 (0.70)	[−0.15, 2.62]	1.75	0.081	3.42	[0.86, 13.67]
	Other	1.09 (1.69)	[−2.24, 4.43]	0.65	0.520	2.98	[0.11, 83.75]
	Staff	0.99 (0.57)	[−0.13, 2.10]	1.75	0.081	2.69	[0.88, 8.20]
	Tasks	1.28 (0.54)	[0.21, 2.34]	2.36	0.019	3.59	[1.24, 10.40]
	Teamwork	-	-	-	-	-	-
Risk Assessment Score	Extreme	−0.17 (0.68)	[−1.50, 1.16]	−0.25	0.81	0.85	[0.22, 3.20]
	High	0.60 (0.35)	[−0.08, 1.28]	1.75	0.08	1.83	[0.93, 3.61]
	Moderate	-	-	-	-	-	-
Course Type	OR	0.34 (1.07)	[−1.77, 2.45]	0.32	0.75	1.40	[0.17, 11.55]
	PACU	1.48 (1.20)	[−0.89, 3.84]	1.23	0.22	4.39	[0.41, 46.51]
	ED	-	-	-	-	-	-

Teamwork, Moderate, and ED remain blank as the reference categories

Intercept = expected odds for an LST from a hospital of average size (average number of beds), that is classified at baseline (i.e., LST coded as teamwork, moderate, ED factors) to be resolved

*β* coefficient, *std*. standard, *CI* Confidence interval, *no*. number, *OR *Operating room, *PACU* Post-anaesthesia care unit, *ED* Emergency department

The multilevel logistic regression model showed an overall statistically significant association between the independent (i.e., hospital size, threat classification, risk assessment scores, course type) and dependent (i.e., resolution status) variables, *F*(12,265) = 2.02, *p* = 0.023. The model explained 61.2% (conditional pseudo-R^2^) of the variance in LST resolution status and correctly classified 82.7% of cases. This suggests the model is a good fit for the data, and the independent variables are likely to have a real, non-random influence on the dependent variable.

Consistent with our univariate analyses, there was no significant association between either of course type or risk assessment score and resolution status.

The results showed that hospital size was a significant negative predictor of threat resolution status (*p* = 0.049). When holding threat classification constant, for every additional 100 beds in a hospital, the odds of an LST being resolved decreased by a factor of 0.804, 95% CI [0.646, 0.999]. That is, when controlling for threat classification, our model suggests that LSTs identified in larger hospitals are less likely to be resolved compared to LSTs identified in smaller hospitals.

In terms of the influence of threat classification, using teamwork as the reference group, the results showed that equipment factors (*p* < 0.001), environment factors (*p* < 0.001), and task factors (*p* = 0.019), were significant predictors of LST resolution status. As all three regression slopes in the logistic regression model were positive, LSTs classified as equipment factors, environment factors, or task factors were more likely to be resolved compared to teamwork factors (when holding hospital size constant). Specifically, the odds of an LST classified as an equipment, environmental, or task factor change by factors of 9.56, 95% CI [3.13, 29.19]; 8.34, 95% CI [2.62, 26.51]; and 3.59, 95% CI [1.24, 10.40], respectively.

Our model found no other significant regression slopes, suggesting that LSTs classified as organisational, staff or other factors when compared to LSTs that are classified as teamwork factors were not significant predictors of LST resolution status.

### Qualitative results

We conducted 15 interviews between October 2023 and October 2024, from 6 nurses, 1 surgeon, 7 emergency medicine physician, and 1 anaesthetist. This represented 1 rural, 9 regional and 5 tertiary hospitals. We identified five themes: influence of threat type on resolution; motivation to resolve the threat; identifying and communicating the threat; agency to implement change; and structures and processes. Illustrative participant quotes are included in Table 3: (#-interview number; S-surgeon; N-nurse; EMP-emergency medicine physician; A-anaesthetist). A more comprehensive table of quotes is provided in Supplementary Table S5.

**Table 3:** Illustrative quotes from each theme

***Influence of threat type***
Yeah and some of the communication stuff- ISBAR- like it’s not a thing anymore really, ‘cause everyone’s got it imbedded. 03, S
There was an issue around the team leader, we have a vest that the team leader wears when committing. There was an issue around the availability of lead aprons for radiology. And there was an issue about the oxygen connectors, are they using different colours for the oxygen, versus medical air. And so all of these were addressed reasonably quickly, because there were easily available solutions. 07, *N*
*It’s easier to follow-up with equipment. It’s easier to follow-up with*,* you know setups*,* or physical objects around you*,* you know? Processes is not that easy. 07*,* N*
*‘Cause I think the main one that has come up every time - how we communicate with Blood Bank. And we’ve talked about ideas and people have talked about it all seemed too hard. 05*,* EMP*
*That’s [Massive Haemorrhage Protocol] one of the big ticket items… there’s lots of people involved. There’s the blood transfusion people… we’ve got to follow their processes*,* but trying to find the best thing that we can do in the hospital. 01*,*N*
*Some things we can’t change*,* the space that we work in. That’s probably one of the biggest safety risks that we have. But*,* thinking how can we optimise that space or how can we improve our use of that space and that was more something that came up actually after this most recent course report that you know. 13*,* EMP*
***Motivation to resolve the threat***
*The people who were in the scenario… we are carrying the momentum of seeing how badly it went – not how badly*,* but how it could have gone better*,* and we’ve got ideas. 05*,* EMP*
*We’re going to have a quick meeting*,* like in the next week*,* with a couple of the key people who were helping to run it. So that we can really focus kind of before it gets a bit stale for us. 05*,* ED*
*It’s just not on their [managers]radar*,* they just don’t have capacity*,* or the bandwidth….despite*,* you know persistently organising meetings and saying we need to meet about this… It takes a fair amount of persistence … that can lead to burnout as well when you don’t have the pickup on it that you want. 09*,* N*
*I think the biggest value we saw*,* apart from the individual level value which is the education training for individual providers was a third party identifying issues that we*,* either we’re aware of*,* Or some new issues*,* or other issues that we hadn’t identified. 04*,* EMP*
***Identifying and communicating the threat***
*First and foremost identifying it [the threat]*,* so that’s the most crucial thing. And then the team all agreeing. Like everyone had to be on the same page as to the severity of the threat. 08*,* N*
*So it was just really identifying the who and how soon that needed to be actioned*,* particularly around the severity of the threat I guess. 12*,* N*
*Then bringing it up at our fortnightly SLT senior leadership team meeting as*,* so that maybe people miss emails or they choose to ignore them or otherwise…. also discussing in a wider. a more interactive kind of forum.13*,* ED*
*It [Massive Haemorrhage Protocol] keeps coming back…as a high risk*,* I then made a nuisance of myself and … got onto the transfusion committee meetings. …so that I could understand what was going on so … I could communicate it. 02*,*N*
*But actually*,* it’s about who you know*,* who you can approach who can you can get on your side? Who are your stakeholders? 01*,* N*
***Agency to implement change***
*Our Chief Medical Officer… was our convenor… that led to a lot of power being able to escalate things high quickly. So that really embedded the concept to the [Hospital] board. 03*,* S*
*There’s a lot of pushback from people… It’s not my job as a nurse educator to tell the anaesthetic group exactly what algorithm we’re gonna follow. 09*,* N*
*I try to cope with*,* I try to manage things*,* effect as much change as I can*,* that I’m capable of*,* because once I ask for somebody else to do … it doesn’t happen. 02*,* N*
*So we’ll just do it as a departmental policy… rather than hospital policy changes. 13*,*EMP*
*I’ve contacted the head of Radiology and asked for just some information around lead aprons… No response. She doesn’t care because it’s not her department. 02*,* N*
*We’re probably easier than some places with it*,* because we don’t need to get buy in from anyone else. As in we’re the whole hospital*,* we’re ED and on the ward*,* and then transporting out. 06*,*EMP*
***Structures and processes***
*Not having anyone specifically employed or allocated to take the list and operationalise it… That’s been really hard… I can’t do it and then some of it gets lost. 11*,* EMP*
*We’ve got a trauma committee*,* deteriorating patient committee…there’s always different people that we can talk to as well. 01*,* N*
*There was an issue around the callout procedure for trauma callouts at our hospital. That was escalated to the clinical board… and that has now resulted in training of admin staff. 09*,* N*
*Sometimes we’ve found that … in terms of chain of command*,* sometimes there are too many steps on that chain… so … just skipping to the top. 03*,* S*

### Influence of threat type on resolution

Participants noted that the nature of safety threats influenced ease of resolution. Improvements were noted in identified problems with team communication, including structured handovers (e.g. between paramedics and ED staff), pre-case briefings, and use of the World Health Organisation Surgical Safety Checklist. Participants noted that improvements were reinforced through repeated training.

Equipment-related knowledge gaps were commonly identified and often readily addressed via in-service training, written guides, and orientation for new staff. Examples included use of defibrillators, blood warmers, and pelvic binders. Role clarity during ED trauma responses improved with the introduction of name-and-role stickers and a team leader vest, though sustainability was challenged by unclear accountability and fading commitment.

Participants noted that implementation of protocols was variably successful. The nationally developed Massive Haemorrhage Protocol (MHP) often conflicted with local practices, causing confusion around requests for blood in major trauma. Respondents highlighted improved collaboration with blood banks and local adaptations as necessary for effective implementation.

Similarly, issues with the Trauma Team call-out protocol led to its revision. Efforts to standardise equipment and clinical pathways met resistance—e.g. aligning paediatric and adult airway trolleys or adopting a single anaphylaxis protocol—primarily due to inter-professional disagreement or perceived justification for variation by individual clinicians. Attempts to streamline emergency drug preparation (e.g. using pharmacy-prepared doses, standardised paediatric protocols) faltered due to interdepartmental impasse, leaving the issue unresolved.

Persistent threats such as insufficient staffing and poorly designed spaces remained largely unaddressed due to financial constraints, according to participants. Some mitigation was achieved by assigning additional roles (e.g. MHP coordinator) or reconfiguring layouts to improve access and workflow. Examples included repositioning equipment, marking emergency items with signage or red tape, and relocating manuals and alarms. These were considered by participants as temporary work-arounds in lieu of comprehensive redesign.

### Motivation to resolve the threat

Participants reported that in situ simulations were highly motivating, offering emotionally engaging experiences including highlighting areas of suboptimal care. This initial motivation was sustained through prompt actions and collaboration in enthusiastic small groups. However, resistance to change—especially when others failed to recognise issues as problems—undermined motivation. Participants reported that persistent advocacy in such contexts could feel burdensome, with some reporting risk of burnout.

Participants reported collaborative change was essential but challenging, particularly when engaging those who had not participated in simulations. Presentations to departments or meetings could help motivate others. While managers were viewed as pivotal to change, they were often difficult to engage due to workload, limited capacity, or lack of resources. Hospital committees were sometimes perceived as detached or even obstructive, prompting participants to focus on localised, intra-department problem-solving. Nonetheless, participants suggested multidisciplinary committees such as Trauma, Quality or Resuscitation Committees could be effective in driving change, although sustaining committee involvement required repeated follow-up and attendance to keep issues visible.

A supportive institutional culture was motivating. Participants described cultural variation: some environments embraced change, others upheld the status quo. External input—such as through NetworkZ training—was also motivating, providing perspective akin to external consultancy. While local insights were valued, external validation could spur action.

### Identifying and communicating the threat

Clear, solution-focused messaging was key to achieving buy-in. This process began with identifying threats during simulations and debriefs, then collaboratively developing solutions, and strategically determining how—and to whom—issues were raised.

Identifying threats required careful observation and skilled debrief facilitation. External perspectives were valued for challenging assumptions and providing inter-hospital comparisons. The structured LST form supported threat identification and categorisation. Small, motivated groups who experienced the threat in simulation were effective in early problem-solving. Involving the broader multidisciplinary team helped refine messaging and co-develop solutions.

Effective interdepartmental communication relied on diplomacy, trust, and relationship-building. Participants built connections with individuals, groups, and committees to understand differing perspectives. Face-to-face interaction was preferred over email. To gain buy-in, communication emphasised actionable steps and targeted the appropriate individuals or groups. Framing issues as patient safety concerns and providing a clear rationale for change were critical to capturing attention and motivating action.

### Agency to implement change

Participants reported that the ability to address patient safety threats relied heavily on the influence of individuals taking responsibility for them. This often derived from their seniority or roles within the hospital. Personal influence was also exerted through existing relationships or proximity, e.g. working in the same office. Participants reported their influence could be limited without explicit responsibility for resolving identified safety threats.

Greater agency was reported within participants’ own departments. Implementing changes across departmental lines was more difficult—for example, ED nurses had limited influence over anaesthetists. Radiology or Blood Bank staff often adhered to their own separate rules. Identifying key decision-makers within each department was essential. Managers held significant agency but were often too overextended to act. Participants from rural hospitals reported that changes could be easier as they worked across the whole hospital.

Agency may reside within specified committees, e.g. the Trauma committee or Quality Committee. However, these committees may not see the issue as important, or they may lack resources for example due to staff vacancies.

### Structures and processes

Participants described varied approaches to managing threats identified during in situ simulations, differing across hospitals. Sometimes convenors assumed personal responsibility or delegation of action; in other cases, information was forwarded to relevant departments.

These informal pathways depended on the convenor’s motivation, skills, and time, as well as the responsiveness of those tasked with follow-up—typically managers or department heads. Convenors often lacked dedicated time and formal mandate. Delegation worked variably; nurse educators could implement training, while department heads were often too overstretched.

The absence of formal roles or processes for addressing threats—particularly those involving multiple departments—made resolution difficult. Local within-department fixes were easier, but implementing cross-department solutions lacked structure. Multidisciplinary committees, such as the Trauma Committee, offered potential avenues for some issues. Where important safety threats could be escalated to the Clinical Board, for example, issues identified with the Trauma Call Out response, action often followed as the Board had the power to implement changes.

Some participants reported using existing online hospital-based incident reporting processes, some of which offered acknowledgement of submissions, though prioritisation by relevant committees was not assured unless actual harm to patients or staff harm had occurred.

### Integration of quantitative and qualitative findings

Quantitative analysis revealed that only 28% of threats were resolved within three months, with resolution significantly influenced by hospital size and threat classification. Qualitative findings provided explanatory depth to these statistical associations. (Table 4) Participants consistently described equipment and environment-related threats as more tangible and actionable, often resolved through straightforward interventions such as signage, training, or workspace reconfiguration. These threats typically fell within the clinician’s sphere of influence, enabling prompt follow-up. In contrast, teamwork and organisational threats required broader cultural or structural changes, which were more difficult to implement due to unclear ownership, interdepartmental boundaries, and limited institutional support.

The lower resolution rates in larger hospitals were explained by qualitative themes highlighting complex hierarchies, fragmented communication, and reduced proximity to decision-makers. Participants from smaller hospitals described flatter structures and greater autonomy, which facilitated faster resolution. The theme of “agency to implement change” emerged as a critical factor, with clinicians reporting that their ability to act was often constrained by role, authority, and institutional processes. Practicality and agency may outweigh formal risk assessments in driving action.

Clinician motivation was high across settings, driven by the emotional impact of simulation scenarios and a desire to improve care. However, without formal structures or support, this motivation often waned, particularly when threats required cross-departmental collaboration or managerial engagement. The absence of clear follow-up pathways and accountability mechanisms further hindered resolution, reinforcing the need for robust governance structures.

**Table 4:** Integration of quantitative and qualitative findings

Quantitative Finding	Qualitative Explanation	Interpretation
Only 28% of LSTs were resolved at 3 months	Clinicians were highly motivated but lacked time, authority, or institutional support to act on threats.	Motivation alone is insufficient; structural support is essential for resolution.
Smaller hospitals had higher resolution rates	Participants in smaller hospitals described flatter hierarchies and easier access to decision-makers.	Organisational size and structure influence the ability to act on safety threats.
Threats related to equipment, environment, and tasks were more likely to be resolved	These threats were seen as tangible, within clinicians’ control, and often had straightforward solutions (e.g., signage, training).	Threats that are concrete and locally actionable are more likely to be addressed.
Teamwork-related threats had the lowest resolution rate	Teamwork issues required cultural change and cross-disciplinary collaboration, which were harder to implement.	Complex, interpersonal or systemic issues face greater barriers to resolution.
No significant association with course type or risk score	Participants did not differentiate resolution efforts based on course type or perceived risk, but rather on feasibility and influence.	Practicality and agency may outweigh formal risk assessments in driving action.
Multilevel regression confirmed hospital size and threat classification as significant predictors	Themes of agency, structures, and processes explained how these factors shaped clinicians’ ability to act.	Statistical associations are grounded in real-world organisational dynamics.

## Discussion

This mixed methods study provides a comprehensive examination of the resolution of latent safety threats (LSTs) identified through in situ simulation across multiple hospital settings. While in situ simulation is widely recognised as an effective tool for uncovering safety threats, our findings reveal a significant gap between identification and resolution, underscoring the need for stronger institutional mechanisms to support follow-through.

Quantitative analysis identified that at three months, 28% of threats were resolved. Hospital size and threat classification were significant predictors of resolution. Smaller hospitals demonstrated higher resolution rates. Threats related to equipment, environment, and tasks were more likely to be resolved than those involving teamwork or organisational factors. Risk score was, counterintuitively, not significantly associated with resolution status.

The qualitative data enriched these findings by illuminating the contextual and interpersonal dynamics that influence resolution. Five interrelated themes emerged: threat type, motivation to resolve, identifying and communicating the threat, agency to implement change, and structures and processes. Clinicians were highly motivated to act on threats identified during simulations, often driven by emotionally salient experiences. However, motivation alone was insufficient. Participants frequently cited barriers such as lack of formal authority, time constraints, and limited institutional support. Higher resolution rates in smaller hospitals were likely due to flatter organisational structures and more direct access to decision-makers. These findings suggest that threats perceived as tangible and within the clinician’s immediate control are more amenable to resolution. It appears that formal risk assessments don’t predict resolution status, suggesting practical considerations override theoretical risk prioritisation in real-world settings.

Agency emerged as a critical factor. Clinicians were more successful in resolving threats when they operated within their own departments or had established relationships with decision-makers. Cross-departmental threats, by contrast, were more difficult to address due to unclear ownership and fragmented communication. The absence of formal roles or processes for managing threats—particularly those requiring multidisciplinary collaboration—was a recurring challenge. Broader change required working across boundaries. Multidisciplinary collaboration led to better solutions but was hindered by the absence of institutional mechanisms to support shared problem-solving. Institutional structures, particularly cross-disciplinary committees, could enable follow-through on identified issues.

The study also highlights the limitations of relying solely on individual initiative. Without formal governance structures, clinicians risk burnout and disengagement. Clinicians were highly motivated to address threats experienced during in situ simulations yet lacked reliable pathways for action. High accountability but low agency is a feature of a work environment with low psychological safety and threatens staff well-being [18]. To fully realise the safety benefits of in situ simulation, robust clinical governance is essential. Establishing empowered multidisciplinary committees (e.g. district-wide trauma committees) to review and act on simulation findings, and increasing regional or national visibility of recurring themes, may promote system-wide learning and improvement. Repeated in situ simulations could test solutions for previously identified safety threats.

### Contribution to existing research

This study adds to existing literature demonstrating the ability of in situ simulation to uncover LSTs and adds a new perspective to the emerging literature on in situ simulation as an effective mechanism for system level improvement in healthcare [19–21].

We provide further information on resolution rates of identified LSTs. Our 3-month resolution rate was lower than that reported by Onge et al. [21] (around 45%) but methodological differences make direct comparison between studies difficult. We add specificity to earlier findings by showing that equipment, environment, and task-related threats are more likely to be resolved than those involving teamwork or organisational issues [5]. These insights align with and extend findings from Patterson et al., [2] Couto et al., [22] and Grace and O’Malley [23], who reported high frequencies of equipment and communication-related threats but did not quantify resolution outcomes.

Our use of multilevel logistic regression to model predictors of resolution provides a more nuanced understanding of how contextual factors, such as hospital size, shape resolution outcomes. This strengthens prior work by Congenie et al. [24] and Mileder et al., [25] who highlighted the importance of structured follow-up and repeated simulation. Combining statistical modelling with thematic analysis offers a richer understanding of the dynamics at play and supports calls for stronger governance and support structures, as advocated by Dadiz et al. [8] and Knight et al. [20]

### Recommendations for practice

Our recommendations include advice for individuals on the one hand and advice for organisations on the other. (Table 5)

**Table 5:** Recommendations for enhancing patient safety through in situ simulation

Recommendation	Description
1. Use in situ simulation strategically	Employ in situ simulation to train staff, assess real-world system performance, and identify threats to the safety of staff and patient safety.
2. Capture threats systematically	Document identified threats using a structured reporting format to facilitate consistent classification and tracking.
3. Involve external expertise	Where possible, include external experts to provide an objective, comparative perspective and uncover issues not apparent to insiders.
4. Build local review processes	Develop hospital-based processes for reviewing identified threats and co-developing practical solutions.
5. Support multidisciplinary collaboration	Establish processes that enable cross-departmental and interprofessional discussion and resolution of safety threats.
6. Empower frontline clinicians	Provide clinicians with the necessary authority, support structures, and resources to take action on identified threats.
7. Ensure institutional accountability	Create dependable systems at both institutional and national levels that hold responsibility for addressing and resolving safety threats.

### Limitations of study

This study has several limitations that should be considered when interpreting the findings.

From 67 initial post-course reports, we were only able to follow up 20 at three months. This is mitigated to some extent by the similarity of the data in the 3-month reports with the original 67 reports (Supplementary Table S4) but we cannot eliminate the possibility of responder bias influencing the reported resolution rate. Our inability to follow-up on safety threat reports could be indicative of the general problem of time poor staff and lack of clear lines of accountability for resolving the threats.

The three-month follow-up period was chosen pragmatically to balance data completeness with feasibility but may have been insufficient for some threats to be fully resolved, particularly those more complex threats requiring structural or cross-departmental changes.

The study relied on self-reported data from local convenors. Independent verification of threat resolution could enhance the objectivity of the outcome measure and it is possible that some threat types may have been less easy to confirm as resolved than others, for example teamwork threats.

The qualitative component involved interviews with 15 convenors, purposively sampled for diversity. While thematic saturation was achieved, the perspectives may not represent all roles, particularly those of department heads or managers, who play key roles in resolution processes. This could be an area for future exploration.

Our risk assessment using HFMEA did not include local staff who may have held different perspectives on risks in their context. Furthermore, the scores were generated to determine the relationship between an external assessment of risk and resolution, rather than information provided to hospital staff to aid prioritisation. This may have contributed to their limited relationship with threat resolution. However, numerical risk scores, while widely used, have notable limitations in guiding risk management. They can be resource intensive, subjective, oversimplify complex clinical contexts, create a false sense of precision, and fail to account for uncertainty in estimates, which can mislead prioritisation and decision-making [26, 27, 28]. While emerging approaches may reduce workload and subjectivity and better capture complex human factors or team dynamics during in situ simulations [20, 29, 30], these concerns underscore the need to better understand how clinicians make decisions on risk.

The study was conducted within the context of the NetworkZ program in Aotearoa New Zealand. While this provides a rich and consistent framework for in situ simulation, the findings may not be generalisable to other simulation programs or healthcare systems with different structures, cultures, or resources. Finally, the study did not quantify the impact of individual convenor characteristics, such as seniority, leadership style, or professional background, on resolution outcomes. These factors may influence agency and effectiveness in addressing safety threats and warrant further investigation.

### Future research priorities

Future research should examine how clinicians’ perceived agency or sphere of influence affects their ability to resolve safety threats. Investigating the relationship between professional autonomy and clinician wellbeing, including job satisfaction and burnout, may offer valuable insights for workforce sustainability and patient safety outcomes.

Future research should also consider extending follow-up periods beyond three months to better capture delayed or staged resolution processes, particularly for complex threats that require structural or policy-level changes.

Further investigation is warranted into the effectiveness of organisational structures, including multidisciplinary committees, governance frameworks, and escalation pathways, in facilitating threat resolution. Additionally, research should examine the enablers and barriers to cross-departmental communication and collaboration, especially in cases where threats span multiple clinical domains.

Simulation-based approaches merit attention as tools for change management. Studies should assess how repeated or follow-up simulations can reinforce learning, evaluate implemented changes, and promote continuous improvement. Comparative research could evaluate the effectiveness of in situ simulation versus traditional incident reporting systems in identifying and resolving safety threats.

Equity and contextual factors should be considered, including how hospital size, geographic location (urban versus rural), and resource availability influence the resolution of clinical threats. Finally, there is a need to develop and validate standardised tools or frameworks that can reliably assess resolution status, impact, and sustainability across diverse threat types and healthcare contexts.

## Conclusion

This study demonstrates that while in situ simulation is a powerful tool for identifying latent safety threats (LSTs), resolution of these threats remains limited. Our findings underscore the importance of aligning institutional processes with the insights of frontline clinicians. Successful threat mitigation depends not only on the nature of the threat but also on the organisational context and the capacity of individuals to act. To fully realise the opportunity presented by in situ simulation to improve patient safety, healthcare systems must move beyond threat identification to actively support resolution—by empowering clinicians, enabling multidisciplinary collaboration, and embedding clear processes for follow-up and accountability.

## Supplementary Information

Supplementary Material 1.

## Data Availability

Non-identifiable original data available on request.
