# Supplementary material for: Resolving latent safety threats identified through in situ simulation: a multicentre mixed-methods study
Source: Adv Simul (Lond). 2025 Dec 8;11:3. doi: 10.1186/s41077-025-00401-y (PMC12801849; doi:10.1186/s41077-025-00401-y)
Supplement: Supplementary file 1 — Supplementary Material 1. [file 41077_2025_401_MOESM1_ESM.docx]

**SUPPLEMENTARY FILES**

**Supplementary Table S1.** *LST reporting tool*.

| **Latent Safety Threats - CLICK ON CELL TO SELECT OPTIONS OR ENTER TEXT** | | |
| --- | --- | --- |
|  | | **Threat 1** |
| **Cause Classification** | **Main** | Drop down selection of the 7 possible main cause classifications (Table S1.1). |
|  | **Secondary** | Dependent on main cause classification selected, drop down of secondary cause classifications (Table S1.1). |
| **Threat Description** | | *Use this section to describe briefly the safety threat that occurred.* |
| **Proposed Actions** | | *List actions required to resolve the identified latent safety threat.* |
| **Who to Action** | | *Provide name and details of person who will be responsible for follow-up of the identified latent safety threat.* |
| **Status at time of course** | | Awaiting initiation; Initiated; Ongoing; Deferred; Complete. |
| **6 Week Follow-Up** | | *Describe any updates to the proposed actions to overcome the identified latent safety threat.* |
| **Status** | | Awaiting initiation; Initiated; Ongoing; Deferred; Complete. |
| **3 Month Follow-Up** | | *Describe any updates to the proposed actions to overcome the identified latent safety threat.* |
| **Status** | | Awaiting initiation; Initiated; Ongoing; Deferred; Complete. |
|  | | |
| **Good Practice Points - CLICK ON CELL TO SELECT OPTIONS OR ENTER TEXT** | | |
|  | | **Point 1** |
| **Good Practice Point** | | *State any identified good practice points.* |
| **Description of Good Practice Point** | | *Use this section to describe briefly the good practice point.* |
| **Follow-up Action Required** | | *List any actions required to take this good practice point forward.* |
| **Who to Action** | | *Provide name and details of who is responsible for identified actions.* |
| **Status** | | Awaiting initiation; Initiated; Ongoing; Deferred; Complete. |

***Note.*** *Instructions in italics were provided to participants within the form. Instructions not in italics filled in automatically or provide a drop-down menu for participants to select a predetermined response. Columns could be added to the form to account for as many latent safety threats as were identified.*

**Table S1.1.** *Drop downs related to main (left column) and secondary cause classifications.*

| Main Cause Classification | Secondary Cause Classifications | | | | |
| --- | --- | --- | --- | --- | --- |
| Teamwork and communication factors | Verbal Communication | Task distribution within team members | Knowing the names of other team members | Seeking help | Other |
| Staff factors | Individual knowledge and skills | Staffing levels and skills mix | Other |  |  |
| Managing the tasks | Task design and clarity of structure | Decision-making (cognitive) aids | Clarity and use of protocols (e.g., MTP, Trauma team) | Other |  |
| Work environment factors | Issues related to medication storage | Design of the work environment | Optimal use of the room | Other |  |
| Equipment factors | Design, availability, maintenance | Other |  |  |  |
| Organisational factors | Policy, standards and goals | Safety culture and priorities | Other |  |  |
| Other factors | Other |  |  |  |  |

**Supplementary Table S2.** *Risk assessment score classification depending on consequence and likelihood scores.*

|  |  | **Consequence** | | | | |
| --- | --- | --- | --- | --- | --- | --- |
|  |  | **Catastrophic (5)** | **Major (4)** | **Moderate (3)** | **Minor (2)** | **Minimal (1)** |
| **Likelihood** | **Almost Certain (5)** | Extreme | Extreme | High | Moderate | Moderate |
|  | **Likely (4)** | Extreme | Extreme | High | Moderate | Moderate |
|  | **Possible (3)** | Extreme | High | Moderate | Moderate | Low |
|  | **Unlikely (2)** | High | High | Moderate | Low | Low |
|  | **Rare (1)** | High | High | Moderate | Low | Low |

**Supplementary Table S3.** *Interview guide.*

| **Topic** | **Interview Questions** |
| --- | --- |
| **Introduction** | *Introduce self.*  *Check understanding of purpose for interview.*  *Check consent for recording.* |
| **Process** | 1. We see from the NetworkZ post course reports that a variety of threats to patient and staff safety have been identified. For example, in your hospital, [insert local examples], and we also see that actions and steps are outlined that aim to resolve these safety issues and events.    1. Can you describe the process during and after a course that you use to identify latent threats?  (**prompts** – who identifies these, when, is there a process for discussion about issues identified?)    2. Can you tell me about your involvement in the resolution of safety issues from the NetworkZ courses at your workplace? 2. When a threat or safety issue is identified through a NetworkZ course how is it managed?    1. Can you give me some examples?  How do you go about deciding what the appropriate course of action is to resolve the threat? *(and who is involved in this?)*    2. Can you tell me about how and why some safety threat resolutions might be escalated? |
| **Facilitators** | 1. What have you found is particularly useful when resolving these safety threats?    1. Can you give me some examples of threats being successfully resolved? |
| **Barriers** | 1. Are there any safety issues or types of events which are more difficult to successfully resolve?    1. Can you give some examples?    2. Why do you think these are particularly difficult?    3. Have you been had any success resolving these types of threats? (why were these instances successful?)    4. Do any particular processes or activities make resolutions easier (chains of communication, quality team involvement, risk registers, etc)?    5. Do some events result in workarounds rather than genuine solutions? Why is this? |
| **Value of NetworkZ programme** | 1. What do you see as the value of reporting latent safety threats? |

**Supplementary Table S4.** *Numbers of courses, number of LSTs, LST categories and resolution status at the initial post-report stage and 3-month follow-up*.

|  | | **Post-Course Report** | **3-month Follow-up** |
| --- | --- | --- | --- |
|  | **No. Hospital Sites** | 24 | 15 |
|  | **No. Courses** | 67 | 20 |
|  | **Total No. LSTs** | 885 | 294 |
|  |  | **N Reports Completed (%)** | **N Reports Completed (%)** |
| **Hospital Demographics** | Tertiary | 34 (50.7) | 6 (30.0) |
|  | Regional | 29 (43.3) | 13 (65.0) |
|  | Rural | 4 (6.0) | 1 (5.0) |
|  |  | **N Hospital Sites (%)** | **N Hospital Sites (%)** |
| **Hospital Demographics** | Tertiary | 5 (20.8) | 3 (20.0) |
|  | Regional | 17 (70.8) | 11 (73.3) |
|  | Rural | 2 (8.3) | 1 (6.7) |
|  | | **N LSTs (%)** | **N LSTs (%)** |
| **Threat Classification** | Teamwork | 238 (26.9) | 77 (27.7) |
|  | Environment | 118 (13.3) | 33 (11.9) |
|  | Equipment | 123 (13.9) | 46 (16.6) |
|  | Staff | 153 (17.3) | 47 (16.9) |
|  | Tasks | 165 (18.6) | 51 (18.4) |
|  | Organisational | 73 (8.3) | 19 (6.8) |
|  | Other | 13 (1.5) | 5 (1.8) |
| **Course Type** | ED | 703 (79.4) | 221 (79.5) |
|  | OR | 132 (14.9) | 30 (10.8) |
|  | PACU | 39 (4.4) | 27 (9.7) |
|  | Radiology | 11 (1.2) | 0 (0.0) |

**Legend**. *No. = number; ED = Emergency Department; OR = Operating room; PACU = Post-Anaesthesia Care Unit.*

**Note.** *Some hospitals completed multiple in-situ simulations and submitted multiple post-course reports. Post-course reports are completed immediately following completion of the in-situ simulation. 3-month follow-ups report on the status LST resolution after a time period of 3 months.*

**Supplementary Table S5.** *Expanded table of participant quotes by theme.(Interview number and profession)*

| ***Influence of threat type*** |  |
| --- | --- |
| *Yeah and some of the communication stuff- ISBAR- like it’s not a thing anymore really, 'cause everyone’s got it imbedded.* | *03, S* |
| *People were getting a bit confused (with Sign Out in the WHO Surgical Safety Checklist) - we had the nurse actually confirming the surgery, rather than the surgeon. So when we got together during the debriefing, …. we found out that the nurses did not quite understand … when you're signing out you actually have to check everything.* | *07, N* |
| *One that was like quite straightforward - we were like sweet, well it’s pretty easy to put that pelvic binder on. You just chuck it on. And then, anyway, it transpired [in NetworkZ course] that actually people didn’t quite know how to put it on ... So that was a good opportunity to like add some written instructions, where it’s kept, nursing education and doctor education. ‘Cause you don’t need like a .. a huge thing on it.* | *05, ED* |
| *So a few of them, so one of them was the stickers identifying key members. There was an issue around the team leader, we have a vest that the team leader wears when committing. There was an issue around the availability of lead aprons for radiology. And there was an issue about the oxygen connectors, are they using different colours for the oxygen, versus medical air. And so all of these were addressed reasonably quickly, because there were easily available solutions, it just needed the supply, sourcing of low-cost equipment. Or just education, and just highlight best practice.* | *07, N* |
| *If it’s something more immediate I think we generally deal with it slightly quicker. So if a, we notice that a crisis book is not in theatre for example we’ll just go and source a new one and replace it immediately.* | *14, A* |
| *We’ve done a lot of work with our rapid infuser, so with nurse education, repeatedly doing simulation using it. Then also we’ve changed the whole way we transfuse people by, when we don’t have to do it massively fast, we use a different device which is a lot more simple. 'Cause we were finding even in the NetworkZ simulations, that half the debrief was always about this Belmont transfuser and how difficult it was to run and that sort of thing.* | *11, ED* |
| *The crisis checklists were absent, or in different locations in some of the theatres. So there were these sort of checklists which were all laminated. And they're now standardised on the back of every single anaesthetic machine.*  *and then there were just specific anaesthetists who took, and techs who took responsibility for that.* | *08, N* |
| *We did a brainstorming session where we kept the equipment in the room and we tried to see if we can optimise the ergonomics - how can we create more room - we moved some equipment, changed how we store some of it .. just created a bit more space.* | *04, ED* |
| *Some things we can’t change, the space that we work in. That’s probably one of the biggest safety risks that we have. But, thinking how can we optimise that space or how can we improve our use of that space and that was more something that came up actually after this most recent course report that you know.* | *13, ED* |
| *If we identify a latent threat that is facility, structural, there’s no chance we’re gonna be able to change that... it costs too much money.* | *10, ED* |
| *However, it’s easier to follow-up with equipment. It’s easier to follow-up with, you know setups, or physical objects around you, you know? Processes is not that easy,*  *like I can’t just go in there and say oh this is what happened during this scenario and I think I would like to change this…You know we don’t have like a trail of that, like maybe I could have done 10 scenarios.* | *07, N* |
| *But, one of them is about our code crimson response. And so because it’s come down from a national level.….[in this hospital] there’s two things called code crimson. .. there’s two steps called the same thing where the blood bank already knows, but doesn’t activate it. It’s been heavily discussed and we just can’t see a way around it because it’s come filtered down [centrally devised process] rather than grown up to us.* | *15, ED* |
| *'cause I think the main one that has come up every time that we need to address is how we communicate with Blood Bank. And we’ve talked about ideas and people have talked about it all seemed too hard. But I think that is one that we will really focus on making an actual action point for us and our department to get a better process with Blood Bank.* | *05, ED* |
| *But that's [MHP] one of the big ticket items. You know, this is there's lots of people involved. There's the blood transfusion people, obviously that are external that are involved. So it's, it's a process driven, kind of, yeah, we've got to follow their processes, but trying to find the best thing that we can do in the hospital.* | *01,N* |
| *So the staffing, which was required, that it seems to be, identifying the underlying cause for that it seems to be difficult in itself. But then the ongoing recruitment, the funding, the process of interviewing. And then retention of staffing, so all of these issues make it difficult because it’s multiple levels. And require organisational intervention, rather than departmental intervention.* | *04, ED* |
| *Now the other issues that are important that keep coming up - lack of staffing and understaffing.. workload pressure .. there’s some things that are out of our control really. And yeah, like a lot of the staffing and the junior staffing, things like that are things that we deal with every day and are probably some of our greatest contributors to errors or to outcome problems, dangers and safety issues….*  *There are issues around the lack of space, the big organisational environment, ergonomics, can be really challenging. We don’t have any money at the moment, there’s no desire to want*  *to buy, or spend any money. We can’t just magic up a bigger resuscitation room, or more space in theatre.* | *04, ED* |
| ***Motivation to resolve the threat*** |  |
| *The people who were in the scenario… we are carrying the momentum of seeing how badly it went – not how badly, but how it could have gone better, and we’ve got ideas.* | *05, ED* |
| *The value is to prevent them [latent safety threats] becoming real harm for real people. So either staff or patients... it’s to try and stop people from breaking their leg by tripping over the wires or – not being able to turn on an ultrasound when someone’s been dying of a stab wound.* | *11,ED* |
| *If you've got good evidence that there’s an issue to start with. If you've got an obvious solution with a why... Because it’s easy to change things, but if you can change something, it might make no difference. Or people won't put it into place unless people actually understand the why.* | *06, ED* |
| *And also the thing I guess that we learnt is that we’re going to have a quick meeting, like in the next week, with a couple of the key people who were helping to run it. So that we can really focus kind of before it gets a bit stale for us.*  *…catch the issues quickly while they’re still kind of fresh in mind and while you’ve still got that almost like emotional engagement of seeing how it played out. And then not chucking it too wide, so really when that issue’s quite fresh thinking okay, I don’t want this to go to a big team. I need this to stick with a small team that’s got the energy, it feels like the ownership of it. And then once you’ve kind of teased out the issue or broken down the issue a little bit more then go okay, who else do we need to involve.* | *05, ED* |
| *I think probably trying to brainstorm a solution at the same time as when you’re [discussing the problem] –*  *Even if you can’t implement that solution. Because no-one likes being brought a problem, but if you bring a problem and some potential solutions* | *11, ED* |
| *So we try to build a kind of a group of interested parties within the department who are looking to progress the stuff. And some people don’t care and they’ve got different interests and that’s fine because everyone’s different in that way. But, you know, there’s a few of us who are interested in kind of governance and quality improvement and patient safety. So we’ve tried to gather all those together in the governance group.* | *13, ED* |
| *What I find is that as a person organising it … it’s really difficult to get time with those people. And follow-up on the latent safety threats … leads to a constant cancellation, or rescheduling of meetings and a burnout. … a lack of progress, and everyone’s schedules are so full … we’re particularly under resourced … we wear a lot of different hats. It takes a fair amount of persistence … that can lead to burnout as well when you don’t have the pickup on it that you want. That report still hasn’t been addressed … rescheduling meetings and then sort of feeling like I'm just being a bother. It’s not me driving it forward, it’s actually them saying oh we need to do something, this is what I'm gonna do. … the manager is really key, because it’s their department.* | *09, N* |
| *“Yeah I'm pretty much involved, I just have to, I'm like a messenger, you know, I'm like a middle person... I would go chasing people... talk to appropriate people... just to make sure that... Because it’s been identified.”* | *07, N* |
| *So I guess I've really picked not so much the low hanging fruit, but I've picked things that I can really, I can make a difference I can carry on and get these things. You know, whether it was depending on how high a risk it was, I did try to prioritize those things* | *02, N* |
| *So there’s actually gotta be the investment that right, we’re gonna run this [NetworkZ] training. We’re gonna get a report about these latent safety threats. And then it’s up to us to do something with them. Because, you know that’s the opportunity, that’s what you're buying into when you get the opportunity to practice your NetworkZ session. Do your team training, have a look at your systems, see what works and what doesn't work. And yeah, it’s a shame that, you know it’s sort of overlooked a lot of the time at this institution. I don’t know what other people’s experiences are like, but I know that we’re under resourced, and under-valued, yeah, it’s a lost opportunity.* | *09, N* |
| *We have that lived experience... and the richness and the discussion.* | *10,ED* |
| *And that just, it’s just not on their radar, they just don’t have capacity, or the bandwidth…..despite, you know persistently organising meetings and saying we need to meet about this.* | *09, N* |
| *I think the biggest value we saw, apart from the individual level value which is the education training for individual providers was a third party identifying issues that we, either we’re aware of, Or some new issues, or other issues that we hadn’t identified. And the biggest value we saw added probably is the direct report from the third part to the quality in the hospital. We thought that’s invaluable, because that’s the biggest value it brings, because it’s not us as a small team in ED identifying these issues. That it’s another party concurring on the same issues and thinking that they're comparing as well with the severity of the threat as well.* | *04, ED* |
| *But, we certainly found it useful to be able to see from an outside perspective what was institutional, what was, you know, thought of as dogmatic or you know locally institutional rather than best practice or benchmarked across kind of the country or Australasia or the world or however it was done.* | *13, ED* |
| *But it brings it to the fore with an external group that helps identify and articulate in a way that's not blaming him or her or one person, it's looking at systems and processes. So I think that I believe that is really beneficial.* | *02, N* |
| *Well hopefully [we are] improving the system and making it a safer environment, safer journey for our patients and for our staff to work in. We’re all striving for perfection in healthcare I guess.* | *14, N* |
| ***Identifying and communicating the threat*** |  |
| *Interviewer: What helps a threat being resolved?*  *Participant: I think first and foremost identifying it, so that’s the most crucial thing. And then the team all agreeing. Like everyone had to be on the same page as to the severity of the threat. And then what helped, you know we’d have to get a point person to actually, you know, to be the one who would resolve it.* | *08, N* |
| *And then I sort of tried to break down the report into, and then kind of send out just specific jobs that were related to specific people’s areas. And then try to get them to bring that back to me.* | *05, ED* |
| *Certainly when you work, you use the word safety and stuff people jump on board with that and they listen.* | *14, A* |
| *And what sort of actions are needed to be taken in regards to, you know, a timeline of that and their responsibility of who was going to action that. So whether that was the charge nurse manager of that department or whether it was a training thing, also whether that educator was going to pick up that side of things. So it was just really identifying the who and how soon that needed to be actioned, particularly around the severity of the threat I guess.* | *12, N* |
| *So I think rather than kind of saying hey Blood Bank that’s a bit of an issue, what do you think, how do you think we could do it better? Actually I think we say hey, we think we should do this, what do you think, should we try this?* | *05, ED* |
| *The second part of that was then bringing it up at our fortnightly SLT senior leadership team meeting as, so that maybe people miss emails or they choose to ignore them or otherwise. So it’s just a second check in, maybe a little bit of accountability. But, also discussing in a wider forum and a more interactive kind of forum where you can see that these are issues that have been identified.* | *13, ED* |
| *And so because it [MHP] keeps coming back, as you know, as a high risk, I then made a nuisance of myself and got on to the got onto the transfusion committee meetings. So I wasn't on the committee, but I got myself invited to the meetings so that I could, so that I could understand what was going on so that I could see where we were up to and I could communicate it.* | *02,N* |
| *I think I remember when I started this job, and my manager at that time gave me a big list of people that I had to go and meet. And, you know, I remember thinking at the time going, this isn't a job. This is like a social thing. But actually, it's about who you know, who you can approach who can you can get on your side? Who are your stakeholders?* | *01, N* |
| ***Agency to implement change*** |  |
| *I think the seniority of the people involved has been something that’s really good. We’ve got, it’s all FMOs who are our convenors at the moment. I mean I’m kind of in two minds about it because I think there’s value in having different types of convenors. But as it stands having a senior medical officer, a convenor, means that they have quite a lot of standing within the departments. And if we say we’ve done this thing and we’ve come across this problem, that has a lot of weight.* | *03, S* |
| *There are some things that I can actually have a profound, or an absolute, or a definite impact... if it involves equipment... that’s not highly expensive.* | *10,ED* |
| *Our Chief Medical Officer … was our convenor… to start with. And that led to a lot of power being able to escalate things high quickly. So that really embedded the concept to the [Hospital] board at that time, that this was something important and if we said something, then it was probably real.* | *03, S* |
| *There’s a lot of pushback from people, or a lack of leadership as to role that out. It’s not my job as a nurse educator to tell the anaesthetic group exactly what algorithm we’re gonna follow.* | *09, N* |
| *I try to cope with, I try to manage things, effect as much change as I can, that I'm capable of, because once I ask for somebody else to do it, once I'm waiting for another person to do something, it doesn't happen.* | *02, N* |
| *I can’t just lift and put it onto somebody else, you know? It’s like a, but basically, it’s an organisational threat so, yeah, I can’t sort of say what are you gonna do about it.* | *11, ED* |
| *So we just did stuff in our department … It effects other specialties who come, but only when their patients are with us. So we’ll just do it as a departmental policy… rather than hospital policy changes.* | *13,ED* |
| *I've contacted the head of Radiology and asked for just some information around lead aprons… No response. She doesn't care because it's not her department.* | *02, N* |
| *Yeah I guess we’re probably easier than some places with it, because we don’t need to get buy in from anyone else. As in we’re the whole hospital, we’re ED and on the ward, and then transporting out.* | *06,ED* |
| *I think out there the teams are so small we work well together all the time. We quite well know each other and because we’re so small we’ve only got that many people. Whereas a bigger trauma that involves ED can involve so many more people.* | *09, N* |
| ***Structures and processes*** |  |
| *So it’s very easy to have lots of things that you think you should implement. And they're not actually implemented because nobody actually does the next step, or educates other people.* | *06, ED* |
| *But it’s not deemed as, they're not gonna just invest more staff into that because it’s been identified as a latent safety threat.* | *09, N* |
| *Not having anyone specifically employed or allocated to take the list and operationalise it... That’s been really hard... I’ve got my fingers in so many pies for this whole thing. And so I can’t do it and then some of it gets lost.* | *11, ED* |
| *They’re generally assigned to whoever, in that faculty, sort of it’s in their area of expertise or area where they sort of operate…..there’s no formal process... we haven’t got dedicated FTE.* | *14, A* |
| *We've got a trauma committee, deteriorating patient committee, force of governance, so there's always different people that we can talk to as well.* | *01, N* |
| *We have like a resus group... responsible maybe for looking at how the equipment’s laid out... we would task them.* | *10, ED* |
| *“There was an issue around the callout procedure for trauma callouts at our hospital. That was escalated to the clinical board... and that has now resulted in training of admin staff.”* | *09, N* |
| *Risk registers definitely are the way... our quality managers are involved... they’re the best ways for us to take it and keep the pressure on.* | *10,ED* |
| *“We have the Safety First system which is an incident reporting system. It is a dog of a computer system... But if it was something that was helpful to put in as a Safety First because they all have to be electronically acknowledged?”* | *11, ED* |
| *Sometimes we’ve found that we just have to, in terms of chain of command, sometimes there are too many steps on that chain. And so going against what the recommended policy is and just skipping to the top.* | *03, S* |

**Legend**. *ED = Emergency Department; N = Nurse; S = Surgeon.*
